# *TIGAR* Is Correlated with Maximal Standardized Uptake Value on FDG-PET and Survival in Non-Small Cell Lung Cancer

**DOI:** 10.1371/journal.pone.0080576

**Published:** 2013-12-10

**Authors:** Xiang Zhou, Wenhui Xie, Qian Li, Yifan Zhang, Jie Zhang, Xiaoping Zhao, Jianjun Liu, Gang Huang

**Affiliations:** 1 Department of Nuclear Medicine, Renji Hospital, School of Medicine, Shanghai Jiao Tong University, Shanghai, China; 2 Department of Nuclear Medicine, Shanghai Chest Hospital, School of Medicine, Shanghai Jiao Tong University, Shanghai, China; 3 Department of Nuclear Medicine, Ruijin Hospital, Shanghai Jiaotong University, Shanghai, China; 4 Department of Pathology, Shanghai Chest Hospital, Shanghai Jiaotong University, Shanghai, China; The University of Hong Kong, China

## Abstract

**Objective:**

Evaluation of ^18^F-FDG uptake value *via* PET is central to current methods of diagnosis and staging of non-small cell lung cancer (NSCLC) due to its ability to evaluate expression levels of key regulators associated with glucose metabolism in tumor cells. Tp53-induced glycolysis and apoptosis regulator (*TIGAR*) is an important P53-induced protein that can inhibit glycolysis; however, there have been few clinical studies on its mechanism. Here we have investigated the relationship between *TIGAR* expression and ^18^F-FDG PET in tumors, along with its relationship with the clinical characteristics of NSCLC.

**Methods:**

We analyzed SUV_max_ in 79 patients with NSCLC through immunohistochemical staining of *TIGAR* and five other biological markers associated with tumor cell glycolysis, in order to evaluate the correlation between their expression and SUV_max_. We also plotted Kaplan-Meier survival curves to assess *TIGAR* expression with the prognosis and survival of patients with NSCLC.

**Results:**

The key findings were as follows: SUV_max_ was negatively correlated with the expression of *TIGAR* (r = −0.31, p<0.01); *TIGAR* expression was correlated with tumor size (p = 0.01), histological type (p<0.01), differentiation degree (p<0.01) and lymph node metastasis(p<0.01) in patients with NSCLC; and the survival time of patients whose *TIGAR* was negatively expressed was significantly shorter than for those whose *TIGAR* was positively expressed (*P* = 0.023).

**Conclusions:**

The expression of TIGAR in primary tumors is significantly correlated with SUV_max_, and low expression of *TIGAR* may predict a worse clinical outcome in patients with NSCLC.

## Introduction

Unlike normal cells, most cancer cells depend on a high rate of glycolysis for energy production during malignant progression. This is known as the Warburg effect and is considered the seventh hallmark of cancer [1]. This effect has been exploited in 18F-fluorodeoxyglucose positron emission tomography/computerized tomography (^18^F-FDG PET/CT) technologies, which has proved highly successful in clinical practice and is widely applied in tumor diagnosis [2], [3].

The maximal standardized uptake value (SUV_max_) determined through PET imaging is a simple and reliable method of evaluating the glucose uptake capacity of tumors *in vivo*. It is defined as the ratio of activity in tissue per unit volume to the activity in the injected dose per patient body weight [4]. Recent reports have demonstrated that the SUV_max_ of primary tumors is correlated with the stage, nodal status, histologic type, differentiation and progression of tumors [5], [6]. Furthermore, a high SUV_max_ has been linked with poor prognosis in cancer patients [7].

Despite these observations, further investigations are required to establish the clinical value of ^18^F-FDG PET/CT imaging for tumor diagnosis, by identifying the metabolic enzymes and abnormal expressions of cancer genes that underlie SUV_max_ changes in cancer tissues. Therefore, many studies have focused on defining the relationship between FDG uptake and the expression of tumor biomarkers, including nuclear- associated antigen Ki-67, cyclooxygenase-2 (Cox-2), vascular endothelial growth factor (VEGF) and the glucose transporter 1 (GLUT1) in relation to lung cancer [4], [8], [9]. To date, these have reported a correlation between SUV_max_ and tumor biomarkers and have contributed to understanding the Warburg effect and identifying tumor-related genes that are associated with the SUV, thereby providing a robust basis for staging, prognosis and personalized treatment of cancers.

Tp53-induced glycolysis and apoptosis regulator (*TIGAR*) is a novel gene related to the glucose metabolism in tumor cells [10]. *TIGAR* inhibits glycolysis by limiting the level of fructose-2, 6-bisphosphate (Fru-2, 6-BP) in the cell by functioning as a Fru-2, 6-bisphosphatase (Fru-2, 6-BPase). It was commonly believed that induction of *TIGAR* led to glycolysis inhibition of the cancer suppressor, *P53* [11]. More recent studies have suggested that *TIGAR* expression increases NADPH levels through activation of the pentose phosphate pathway (PPP), thereby promoting antioxidant function which reduces reactive oxygen species (ROS)-associated apoptosis and enhances cancer cell survival [12]. However, these studies were conducted on a limited number of cell lines through intervening transient expression experiments; therefore, it remains to be established whether *TIGAR* expression is correlated with SUV on FDG-PET and tumor prognosis in clinical practice, by acting as a suppressor gene of glucose metabolism.

The purpose of this present study was to assess the correlation between SUV_max_ and the expression of selected markers associated with tumor metabolism, namely, glucose transporter 1(*GLUT1*), hexokinase 2 (*HK2*), pyruvate kinase M2 (*PKM2*), lactate dehydrogenase A (*LDHA*), protein kinase B (*AKT*) and *TIGAR*. In addition, the relationship between the *TIGAR* expression and prognosis in non-small cell lung carcinoma (NSCLC) was investigated. We selected 79 NSCLC cases for this study, with the aim of identifying novel biological indictors for clinical diagnosis and personalized treatment options, by analyzing the relationship between SUV and key regulatory factors in glucose metabolism.

## Patients and Methods

### Ethics Statement

The Human Investigation Ethical Committee of Shanghai Jiao Tong University affiliated Renji Hospital and Shanghai Chest Hospital approved this study. All procedures involving human specimens were performed with written informed consent according to the Declaration of Helsinki.

### Study Population

This was a retrospective study of all patients who were confirmed to have NSCLC based on histopathological finding and underwent surgery after ^18^F-FDG PET/CT between December 2006 and December 2009 at Shanghai Jiaotong University affiliated Renji Hospital and Shanghai Chest Hospital. Eligibility criteria were (1) without receiving chemotherapy/ radiotherapy before PET/CT scanning; (2) tumor pathology of NSCLC (excluded benign lung lesions and small cell lung cancers); (3) complete case records; (4) performed PET/CT scanning no more than 2 weeks prior to surgery; (5) available tissue specimen for IHC staining. Finally, 79 patients (50 male and 29 female) with a median age of 61 (range, 30–79 years) were evaluated in this study. All clinical and pathological findings were reviewed from three hospitals in Shanghai, China (Renji Hospital, Shanghai Chest Hospital, Ruijin Hospital).

### PET/CT imaging

A dedicated whole-body PET/CT tomography (SIMENS) was used for all PET/CT imaging. Image acquisition was performed with an integrated PET/CT device. Immediately after CT scanning, PET was performed to cover the identical axial field of view. PET-image data sets were reconstructed iteratively with segmented correction for attenuation using the CT data. For semi-quantitative analysis of the ^18^F-FDG uptake, irregular regions of interest (ROIs) were placed over the most intense area of ^18^F-FDG accumulation. The maximum SUV (SUVmax) was calculated using following formula: maximum pixel value with the ROI activity (MBq/kg)/(injected dose [MBq]/body weight [kg]). Two experienced nuclear medicine physicians, blinded to the clinical history, independently evaluated the PET images and reached a consensus on all image results.

### Immunohistochemical Staining

Immunohistochemical analyses were performed on paraffin-embedded lung cancer tissues. After microtome sectioning (4 µm slices), the slides were processed for staining. All primary antibodies (*TIGAR*, *GLUT-1*, *HK-2, PKM-2, LDHA, AKT*) were purchased from Abcam. Sections were assessed using a light microscope (BX51TR, Olympus, Japan) by 2 independent observers. The expression of each marker protein was examined and statistical software (DFC320 CCD system, Leica, Germany) was used for semi-quantitative analysis of IHC staining. The slides were scored for intensity of staining (0 to 3) and the percentages of cells with scores of 0 (0%), 1 (1% to 9%), 2 (10% to 49%), and 3 (50% to 100%) were determined. The immunohistochemistry (IHC) score (0 to 9) was defined as the product of the intensity and percentage of cells. Protein expression was judged as positive when the IHC score was greater than or equal to 4. All IHC results were evaluated by 2 experienced observers who were blind to the condition of the patients. Where discrepancies occurred by 2 readers, the 2 readers reached a consensus.

### Statistical Analysis

Continuous variables were analyzed by the Student's t test, and the results were expressed as mean ± standard deviation. Dichotomous variables were analyzed by the x2 test. We used Spearman rank correlation to examine the association between SUVmax and protein expression. To explore the association between recurrence-free survival and TIGAR expression, a Kaplan-Meier survival analysis was performed. Two- sided p values of less than 0.05 were considered to be statistically significant. All analyses were performed using SPSS software, version 16.0 (SPSS Inc, Chicago, IL).

## Results

### Clinical and pathological characteristics of patients in relation to SUV_max_

The clinical and pathological characteristics of the 79 patients with NSCLC in this study are illustrated in Table 1. The patients were aged between 30 and 79 years (mean; 60.85±10.40 years) and included 50 males and 29 females. The mean SUV_max_ was 7.24±4.58, the maximum, minimum and median values were 24.1, 0.7 and 6.6 respectively, and 10 patients had a SUV_max_ <2.5. Statistical analysis showed that there were no significant differences in SUV_max_ between patients ≥60 years old and patients <60 years old or between male and females patients.

**Table 1:** Clinical and pathological characteristics in relation to SUV_max_ in patients with NSCLC.

Characteristic	No. patients	SUVmax	*P*-value
Age			
60 years	39	6.85±3.81	0.58
60 years	40	7.42±4.23	
Gender			0.37
Male	50	7.65±4.27	
Female	29	6.54±4.84	
Tumor size (cm)			<0.01
≤3.0	54	5.62±3.82	
>3.0	25	10.7±4.18	
Pathology			<0.01
Squamous	24	10.60±4.73	
Adenocarcinoma	55	5.94±4.04	
Tumor differentiation			<0.01
Well	13	4.4±3.1	
Moderate	34	7.2±4.2	
Poor	32	12.7±4.1	
Pathological N stage			<0.01
N0	55	6.33±4.60	
N1	24	9.31±3.89	
Outcome			<0.01
Alive	42	6.13±3.89	
Dead	20	9.66±4.15	

SUV_max_  =  maximal standardized uptake value.

However, there were significant differences in SUV_max_ between patients with tumors >3 cm and those with tumors ≤3 cm in size. In addition, SUV_max_ was statistically different between patients with well, moderate and poorly differentiated tumors; between patients with squamous carcinoma and adenocarcinoma and between patients with pathological stage N0 and N1 tumors (p<0.01).

### Relationship between FDG-PET and immunohistochemical scores of proteins involved in glucose metabolism in tumor cells

We carried out immunohistochemical (IHC) analysis in order to assess the correlation between PET SUV_max_ and the expression of selected proteins associated with glucose metabolism in tumor cells according to their IHC staining scores.

**Figure 1:**
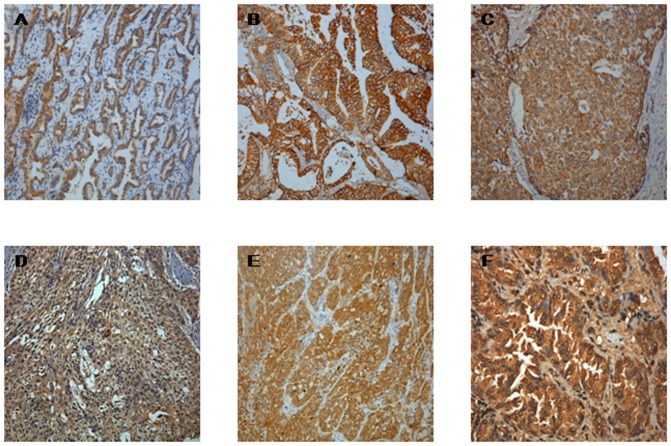
Immunohistochemical analysis showed positive staining. A: *TIGAR*, B: *GLUT*, C: *HK2*, D: *PKM*, E: *LDHA*, F: *AKT* (magnification, ×400).

The results are shown in Figure 1 and presented in Table 2. SUV_max_ of FDG showed a negative correlation with TIGAR expression (r = −0.31, p<0.01). In addition, SUV_max_ was significantly correlated with expression levels of *GLUT1*(r = 0.37, p<0.01). However, SUV_max_ was weakly correlated with *PKM2* and not correlated with glucose metabolism genes, *LDHA, HK2* or oncogenes, *AKT*.

**Table 2:** Pearson correlation coefficients and p-value between the immunohistochemistry (IHC) staining scores for genes expression associated with glucose metabolism in tumors with SUV_max_.

Factor		SUVmax	
	correlation coefficients		*P*-value
*TIGAR* expression	0.31		<0.01
*GLUT-1* expression	0.37		<0.01
*HK-2* expression	0.08		0.45
*PKM-2* expression	0.21		0.06
*LDHA* expression	0.09		0.39
*AKT* expression	0.04		0.71

There was a significant difference in SUV_max_ between patients with positive and negative expression of *TIGAR* (*P*<0.01). The mean value of SUV_max_ was 8.29±3.64 in the TIGAR negative cases and 6.31±3.14 in the TIGAR positive cases. Similarly, SUV_max_ was significantly different between patients with positive and negative expression of *GLUT1* (*P*<0.01). The mean value of SUV_max_ was 6.09±3.60 in the *GLUT1* negative cases and 8.83±5.33 in the *GLUT1* positive cases (Table 3).

**Table 3:** Correlation between TIGAR or GULT-1 expression and SUV_max_.

Factor	No. patients	SUVmax	*P*-value
*TIGAR* expression			<0.01
Negative	37	8.29±3.64	
Positive	42	6.31±5.14	
*GLUT-1* expression			<0.01
Negative	46	6.09±3.60	
Positive	33	8.83±5.33	

We further analyzed the impacts of the different expression level of *TIGAR* and *GLUT1* on glucose uptake (Table 4). The maximum SUV_max_ (mean, 10.20±3.40) was observed in patients with negative expression of *TIGAR* and positive expression on *GLUT1*. Conversely, the minimum SUV_max_ (mean, 5.47±3.89) was observed in patients with positive expression of *TIGAR* and negative expression of *GLUT1*.

**Table 4:** Correlation between expression of *TIGAR* and *GLUT-1* with SUV_max_, based on immunohistochemical (IHC) score.

		*TIGAR* (IHC score)	
	No. patients	No. patients	
		Negative	positive
	Negative	6.82±3.17	5.47±3.89
GLUT-1	46	21	25
	Positive	10.20±3.40	7.54±6.51
	33	16	17

### Relationship between TIGAR expression and the biological characteristics and staging grade of NSCLC tumors

Although *TIGAR* is closely linked to glucose uptake in tumor tissues, there have been few reports on its relationship with the biological characteristics of tumor tissues. Therefore we analyzed *TIGAR* expression levels in tissue samples from 79 patients with NSCLC in relation to age, gender, tumor size, histological type, histological degree and tumor staging. The results are presented in Table 5. These showed that the difference between *TIGAR* expression in respect of patient gender or age (<60 or ≥60 years) was not significant. Conversely, the difference was significant between *TIGAR* expression in patients with respect to tumor size (≤3 or >3 cm), histological type (squamous or adenocarcinoma), differentiation degree (well or poor) and staging (N0 or N1).

**Table 5:** The relationship between *TIGAR* expression and tumor characteristics, based on immunohistochemical (IHC) score.

	TIGAR (IHC score)		
Characteristic	No. patients (n = 79)		
	Negative	Positive	*P*-value
Age			0.22
<60 years	21	18	
≥60 years	16	24	
Gender			0.09
Male	27	23	
Female	10	19	
Tumor size (cm)			0.01
≤3	20	34	
>3	17	8	
Pathology			<0.01
Squamous	18	7	
Adenocarcinoma	19	35	
Tumor differentiation			<0.01
poor	21	11	
well	16	31	
Pathological N stage			<0.01
N0	19	36	
N1	18	6	

### The relationship between TIGAR expression with survival and prognosis of patients with NSCLC

**Figure 2:**
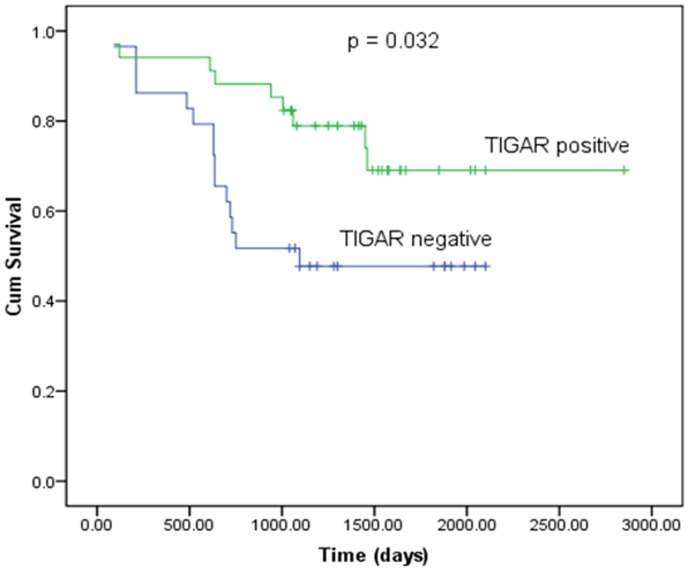
Kaplan-Meier recurrence-free survival curve according to *TIGAR* expression in patients with NSCLC. The survival time of patients with negative expression of *TIGAR* is significantly shorter than those with positive expression of *TIGAR*.

In order to assess the impact of *TIGAR* expression on patients' survival, we plotted Kaplan-Meier survival curves for patients with positive expression and negative expression of *TIGAR* (Figure 2). The results showed that the survival time of patients whose *TIGAR* was negatively expressed was significantly shorter than for those whose *TIGAR* was positively expressed (*P* = 0.023 according to the log-rank test).

## Discussion

Lung cancer has the highest incidence rate of human malignancies and is the leading cause of cancer mortality worldwide. Patients with NSCLC account for 80 to 85% of all patients with lung cancer; therefore, early diagnosis and targeted treatments are critical to improving survival rates of lung cancer patients [13]. In this respect, FDG-PET molecular imaging techniques have provided major advances in clinical diagnosis of lung cancer. In accordance with previous reports, our study showed that SUV_max_ was closely correlated with tumor size, differentiation degree and pathological type in NSCLC patients. In addition, patients with higher SUV_max_ had an increased probability of lymph node metastasis and a worse prognosis. High SUV_max_ is correlated with an increase in glucose metabolism and the Warburg effect in tumor cells. It is generally acknowledged that the Warburg effect is associated with abnormal expression of glucose metabolism enzymes (*GLUT1, HK2,PKM2, LDHA*), oncogenes (*Ras, MET*), signal transduction kinases (*AMP, AKT*) and mitochondrial proteins (IDH3, COX2), as well as a hypoxic microenvironment in which the expression level of hypoxia-inducible factor 1 (HIF-1) increases [14]–[16]. However, the mechanisms underlying the Warburg effect are not yet fully understood.

In our present study, the expression levels of seven metabolic enzymes associated with glucose metabolism in tumor cells were compared with SUV_max_ in 79 patients with NSCLC. Our results showed that SUV_max_ of FDG uptake was significantly correlated with expression levels of *GLUT1* and *TIGAR*, weakly correlated with *PKM2* and not correlated with glucose metabolism genes, *LDHA, HK2* or oncogenes, *AKT*.

Previous reports have suggested that *GLUT1* has an important role in ^18^F-FDG uptake in patients with lung cancer, soft tissue sarcoma and pancreatic cancer [9], [17], [18]. Conversely, Tohma et al. reported low correlation between ^18^F-FDG uptake and *GLUT1* expression in esophageal cancer [19]. Our data demonstrated that *GLUT1* expression was significantly associated with high SUV_max_, supporting earlier reports that *GLUT1* expression contributes to ^18^F-FDG uptake in NSCLC patients.

Notably, this study has provided the first evidence of a correlation between *TIGAR* expression and SUV_max_ on FDG-PET in patients with NSCLC. Our data showed that *TIGAR* expression is negatively correlated with tumor SUV_max_. Furthermore, SUV_max_ was highest in patients who had positive expression of *GLUT1* with negative expression of *TIGAR*. This further demonstrated that *TIGAR* involved in glycolysis in lung cancer and *TIGAR* inhibition could remove the negative regulation of glucose metabolism, thereby increasing glycolysis in tumor cells.

Many experiments that have been performed at the cellular level have confirmed that *TIGAR* can inhibit glycolysis. This is consistent with our clinical observations. It is generally considered that the glycolysis mechanism induced by *TIGAR* bears close similarity to that of the bisphosphatase (FBPase) domain of 6-phosphofructo-2-kinase/fructose-2, 6-bisphosphatase (PFK-2/FBPase-2), in that *TIGAR* expression leads to a reduction in fructose-2, 6-bisphosphate (Fru-2, 6-BP) levels, thereby suppressing glycolytic flux and tumor growth. Fru-2, 6-BP is a potent allosteric regulator of PFK1, an early enzyme in the glycolytic pathway. Furthermore, the decrease in Fru-2, 6-BP levels may lead to decreased GK (the hepatic isoform of hexokinase) and increased G6Pase levels [20], providing an additional means by which *TIGAR* can negatively regulate glycolysis.

Activation of glycolysis is not only beneficial to tumor metabolism for energy production and proliferation, but can also activate multiple transcription factors associated with oncogenes and tumor development, such as sterol regulatory element binding protein (SREBP) which is involved in fatty acid synthesis [21]. Therefore, these glycolytic genes have also been the focus of studies on tumor development. It is increasingly apparent that *TIGAR* may play other significant roles in controlling processes involved in human malignancies; however, the mechanisms remain to be fully understood. It has been suggested that *TIGAR* inhibits tumor growth by acting in a similar manner to the tumor suppressor *P53*. López-Guerra et al. found that high expression of *TIGAR* was positively correlated with chronic lymphocytic leukemia (CLL) sensitivity to Fludarabine, which can induce apoptosis [22]. Madan et al. showed that *TIGAR* could stabilize the retinoblastoma protein and E2F transcription factor 1 (Rb-E2F1) complex leading to cell cycle arrest and inhibition of tumor growth [23]. Similarly, Hesegawa found that high expression of *TIGAR* promoted cell cycle arrest and cellular senescence, whereas a *TIGAR* knockout acted to reduce apoptosis [24]. However, a conflicting mechanism proposed by Bensaad et al. suggested that *TIGAR* inhibited glycolysis at the cellular level while activating the PPP, which inhibited ROS production and tumor cell apoptosis and autophagy; thereby promoting tumor growth in U2OS and RKO cell lines [12].

The majority of studies on *TIGAR* have been performed at the cellular level with only a few reports on clinical studies. Therefore, we conducted this study to compare *TIGAR* expression with the clinical characteristics of tumors, and its relationship to prognosis and survival in patients with NSCLC. We demonstrated that *TIGAR* expression was significantly higher in lung adenocarcinoma tissue than in squamous carcinoma (*P*<0.01), which is consistent with observations of lower SUV in lung adenocarcinoma. We found that well-differentiated NSCLC had higher expression levels of *TIGAR* compared to poorly differentiated NSCLC, indicating increased malignancy. In addition, we showed that patients with negative expression of *TIGAR* were more likely to develop lymph node metastasis than those with positive expression of *TIGAR*. These indicated that *TIGAR* expression was closely correlated with prognosis. Survival curve analysis showed that the survival rate declined significantly in NSCLC patients who had low levels of *TIGAR* expression (*P = *0.023) compared those with high levels. These findings, based on clinical studies of NSCLC tissues, suggest that quantification of TIGAR protein levels may help predict prognosis of patients with NSCLC and cancer treatment targeting TIGAR might become feasible in the future. We have proposed to conduct a trial of a *TIGAR* activator for the treatment of lung cancer in PET-positive mice with the aim of developing potential gene targeted therapies for lung cancer.

In conclusion, the expression of *GLUT1* and *TIGAR* was correlated to SUVmax in NSCLC. This is the first report that describes a significant correlation between the expression of *TIGAR* and SUV_max_, and showed that low expression of *TIGAR* in primary NSCLC tumors was strongly correlated with a worse clinical outcome. These findings indicate that TIGAR may help predict prognosis of cancer patients and facilitate selection of patients for targeted therapies involving *TIGAR* in NSCLC. Further studies are required to identify lung cancer patients who are suitable for *TIGAR*-targeted therapy based on pre-evaluation of FDG uptake.
